# Molecular Epidemiology and Seroepidemiology of Oz Virus Infection in Ticks and Wild Boars in Ibaraki Prefecture, Japan

**DOI:** 10.3390/microorganisms13112421

**Published:** 2025-10-22

**Authors:** Shuichi Osawa, Hirokazu Kimura, Sakurako Abe, Noriko Nagata, Eri Ueno, Hideki Ebihara, Ryusuke Kimura, Tetsuya Furuya

**Affiliations:** 1 Ibaraki Prefectural Institute of Public Health, 993-2 Kasaharacho, Mito-shi 310-0852, Ibaraki, Japan; sh.oosawa@pref.ibaraki.lg.jp (S.O.);; 2 Cooperative Department of Veterinary Medicine, Faculty of Agriculture, Tokyo University of Agriculture and Technology, Harumicho, Fuchu-shi 183-0057, Tokyo, Japan; 3 Department of Health Science, Graduate School of Health Sciences, Gunma Paz University, Takasaki-shi 370-0006, Gunma, Japan; 4 Advanced Medical Science Research Center, Gunma Paz University, Takasaki-shi 370-0006, Gunma, Japan; 5 National Institute of Infectious Diseases, Japan Institute for Health Security, 1 Chome-23-1 Toyama, Shinjuku 162-8640, Tokyo, Japan

**Keywords:** Oz virus (*Thogotovirus ozense*), molecular epidemiology, seroepidemiology, tick, wild boars

## Abstract

Oz virus (OZV), an emerging negative-sense single-stranded RNA virus classified under the family *Orthomyxoviridae* and genus *Thogotovirus*, was first isolated from *Amblyomma testudinarium* ticks in Ehime Prefecture, Japan, in 2013. Moreover, a single fatal case in an elderly individual, suspected to be associated with OZV infection, was reported in Ibaraki Prefecture in 2023. Given these circumstances, this study was conducted to investigate the molecular epidemiology and seroepidemiology of OZV in Ibaraki Prefecture, Japan. From April to November 2023, a total of 2430 ticks were collected at 19 sites. The OZV RNA was detected in one *A. testudinarium* nymph. Additionally, among 934 wild boar serum samples collected between 2019 and 2023, one sample tested positive for OZV RNA. Neutralizing antibody assays revealed that 243 samples (26.0%) obtained from wild boars were seropositive, indicating widespread exposure among wild boars. Antibody prevalence and titers were highest in the central–western mountainous region, suggesting an active transmission hotspot. Sequence analysis of the OZV viral RNA detected from one tick sample and one wild boar serum revealed that a 212 bp fragment of segment 4 and a 261 bp fragment of segment 5 were 100% identical to a human-derived strain isolated in the same prefecture, suggesting the circulation of a single viral lineage within the local environment. These findings represent the first report demonstrating the circulation of OZV in the natural environment in Ibaraki Prefecture, implicating *A. testudinarium* as the principal vector and wild boars as a potential source of OZV infection These results suggest that OZV should be considered a potential emerging zoonotic pathogen. Further seroepidemiological studies among residents are warranted to assess the risk of human infection in the region.

## 1. Introduction

Oz virus (OZV) is an emerging RNA virus classified within the family *Orthomyxoviridae* and the genus *Thogotovirus* [1]. This pathogen possesses a six-segmented, single-stranded, negative-sense RNA genome, approximately 10.6 kb in length. OZV was first isolated and identified from *Amblyomma testudinarium* ticks collected in Ehime Prefecture, Japan, in 2013 [1]. Serological surveys of wild animals, including Japanese macaques (*Macaca fuscata*), Japanese wild boars (*Sus scrofa leucomystax*), sika deer (*Cervus nippon*) and Asian black bears (*Ursus thibetanus*) suggest that OZV is distributed in certain regions of Japan [2]. Although the clinical history of infection remains unclear, a serological study conducted on domestic hunters in Yamaguchi Prefecture, Japan (located in the western region of the country), reported that two out of 24 individuals tested positive for anti-OZV antibodies, as determined by Plaque Reduction Neutralization Test (NT-Ab) [3]. While the pathogenicity of OZV remains uncertain, a fatal case suspected to have been caused by OZV infection was reported in early summer 2022 in Ibaraki Prefecture, Japan [4]. This suggests that OZV may be highly pathogenic and potentially fatal in humans, although such cases may be rare.

Ibaraki Prefecture is located in the northeastern part of the Kanto region, Japan, adjacent to the densely populated Tokyo metropolican area and, therefore, infectious diseases of its people have potential of public health impact on the people in the Tokyo area. The prefecture is approximately 750 km away from Ehime Prefecture, where OZV was first identified [1] but mode of transfer of the virus between the prefectures is clear yet. In 2025, a serological survey of OZV targeting companion animals (dogs and cats) was conducted in Ibaraki Prefecture [2]. However, all the animals tested negative for antibodies, and the presence and prevalence of OZV in the natural environment remained unknown. Therefore, this study aims to investigate the molecular epidemiology of OZV in various tick species and conduct a seroepidemiological survey of wild boars in Ibaraki Prefecture in 2023.

## 2. Materials and Methods

### 2.1. Tick Collection from Vegetation and RNA Extraction

From April to November 2023, ticks were collected using the flagging method, in which a 1 m^2^ white cloth was dragged over vegetation and the attached specimens were retrieved. Collected ticks were morphologically identified to the species level. For each sampling date and location, ticks of the same species were grouped into larvae, nymphs, and adults, and pooled at a maximum of 15 larvae, 10 nymphs, or 5 adults per pool, selected randomly. The tick specimens were frozen and homogenized in liquid nitrogen using a Biomasher II (Nippi, Tokyo, Japan). Total RNA was then extracted by adding 800 μL of Isogen II (NIPPON GENE, Tokyo, Japan) according to the manufacturer’s protocol.

### 2.2. Collection of Wild Boar Serum and RNA Extraction

From April 2019 to December 2023, serum samples were collected from 934 wild boars captured in Ibaraki Prefecture. These serum samples were collected and stored by the Northern Ibaraki Prefectural Livestock Hygiene Service Center as part of the monitoring program for classical swine fever outbreaks in the prefecture. The capture location, sex, and body length of each wild boar were recorded by hunters. The capture locations of the wild boars were plotted on an Ibaraki Prefecture map created using QGIS [5], based on the National Land Numerical Information dataset [6]. Total nucleic acids were extracted from the serum samples using the Viral Total Nucleic Acid Purification Kit (Promega Corporation, Madison, WI, USA).

### 2.3. Detection of OZV RNA

Screening for OZV RNA was performed using reverse transcription-quantitative PCR (RT-qPCR) targeting segment 5 (*nucleoprotein* gene; *NP* gene) with the TaqMan™ Fast Virus 1-Step Master Mix (Applied Biosystems, Foster City, CA, USA) as previously reported [2,4]. Samples that tested positive in the screening were further analyzed using conventional RT-PCR targeting segment 4 (*glycoprotein* gene; *GP* gene) and segment 5 with the PrimeScript II High Fidelity One Step RT-PCR Kit (Takara Bio, Shiga, Japan). The *GP* gene (segment 4) may reflect antigenic diversity and host adaptation [7], while the *NP* gene (segment 5) may offer a more conserved marker for stable phylogenetic positioning [8]. Nested PCR was performed for using the primer sets (Table S1) and PrimeSTAR GXL DNA Polymerase (Takara Bio, Shiga, Japan). The primers used for these analyses were designed using the Primer-BLAST (NCBI, https://blast.ncbi.nlm.nih.gov/Blast.cgi, accessed on 19 October 2025). For RT-qPCR and conventional RT-PCR, extracted RNA from the OZV EH-8 strain was used as the positive control, and nuclease-free water was employed as the negative control. The presence or absence of amplification was confirmed using an Agilent 2100 Bioanalyzer (Agilent Technologies, Santa Clara, CA, USA), which provides more consistent sizing, sensitivity and data traceability than standard gel electrophoresis. The primers and probes used are listed in Table S1. Samples that tested positive for conventional RT-PCR in both segments were considered OZV RNA-positive. We used PooledInfrate to calculate the OZV infection rate per 1000 ticks from the pooled tick specimens [9]. To minimize the risk of cross-contamination, pre-PCR procedures, including reagent preparation and template addition, were conducted in a designated clean area, while post-PCR processes involving amplification products were performed in a physically separate space with a unidirectional workflow. Reagents were prepared as master mixes prior to the addition of templates, and aliquoted to avoid repeated freeze–thaw cycles. Positive controls were stored separately and handled only at the final stage of the experimental procedure. Moreover, the RT-qPCR assay employed in this study was performed previously described [2,4]. However, the detection sensitivity of the method was not described in those reports. Therefore, using the synthetic RNAs for the genes (Fasmac, Kanagawa, Japan), the minimum RNA copy numbers detected with the assays were estimated to be 5 copies for the RT-qPCR, and 10 and 100 copies for the nested and the regular RT-PCR assays, respectively. The detailed data, including standard curves and amplification profiles, are provided in Supplementary Figures S1 and S2.

### 2.4. Sequence Analyses

PCR products were purified using Merck Amicon Ultra-0.5 Centrifugal Filter Units (Merck, Darmstadt, Germany). The purified products were sequenced using the BigDye Terminator v3.1 Cycle Sequencing Kit (Applied Biosystems, Norwalk, CT, USA) and analyzed on a 3500xL Genetic Analyzer (Applied Biosystems, Norwalk, CT, USA). The obtained sequences were aligned with orthomyxovirus sequences retrieved from GenBank using Clustal W method in MEGA 6 [10]. Phylogenetic trees were constructed using the maximum likelihood (ML) method, and their statistical significance was assessed using the bootstrap method with 1000 replicates.

### 2.5. NT-Ab Against OZV

Serum samples collected from wild boars were subjected to the NT-Ab against OZV method. A mixture of 50 μL of heat-inactivated serum diluted at 1:10 and 50 μL of the OZV EH-8 strain (50 PFU) was incubated at 37 °C for 60 min. The supernatant was removed from Vero cells cultured in 24-well plates, and the mixture was added, followed by incubation at 37 °C for 1 h. The infected cells were then overlaid with a culture medium containing 1% methylcellulose. After a four-day incubation, cells were fixed with formaldehyde and stained with methylene blue. Serum samples that reduced plaque numbers by 80% were considered antibody-positive. For antibody-positive serum samples, NT-Ab was performed with final dilutions ranging from 1:20 to 1:320. The highest dilution factor at which plaque numbers were reduced by 80% was defined as the neutralizing antibody titer. Statistical analyses of the geometric mean antibody titer (GMT) and antibody positivity rate were conducted using JMP^®^ 18 (JMP Statistical Discovery LLC, Cary, NC, USA). We also calculated their confidence intervals (CI). The Kruskal–Wallis test was used for GMT comparisons, while Fisher’s exact test was used to evaluate differences in antibody positivity rates.

## 3. Results

### 3.1. Detection of OZV RNA from Ticks

A total of 2430 ticks belonging to three genera and seven species, were collected (Table 1). Five-hundred thirty tick pooled samples were subjected to RT-qPCR to detect OZV RNA. As a result, one *A. testudinarium* nymph, collected in May 2023 from the central-western region of the prefecture was tested positive for OZV RNA (Ct value: 31). The sample was also confirmed to be positive by conventional RT-PCR targeting segment 4 (GP) and segment 5 (NP). The positive rate of OZV RNA in *A. testudinarium* nymphs was estimated to be 3.65 (95% CI: 0.21–17.47).

### 3.2. Detection of OZV RNA in Wild Boar Serum

Among 934 wild boar serum samples, one tested positive for OZV RNA by RT-qPCR (Ct value: 36). This sample was also confirmed to be positive by conventional RT-PCR targeting segment 4 (GP) and segment 5 (NP). The positivity rate of the OZV RNA in wild boar serum samples was estimated to be 0.11%. The infected wild boar was a 20 kg, 80 cm-long male, captured in the central-western region of the prefecture in June 2021.

### 3.3. Sequence and Phylogenetic Analyses of OZV Strains

**Figure 1:**
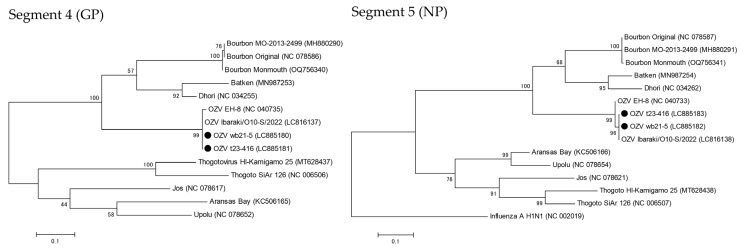
Phylogenetic trees of segment 4 (glycoprotein, GP) and segment 5 (nucleoprotein, NP) of OZV detected strains (OZV t23-416 and OZV wb21-5) and other Orthomyxoviridae viruses. Bootstrap values are shown next to each node. The positions of OZV t23-416 and OZV wb21-5 are highlighted with black dots.

The nucleotide sequences of the OZV strains detected from a tick (t23-416) and a wild boar (wb21-5) were analyzed. The segment 4 (212 bp) and segment 5 (261 bp) sequences of strains t23-416 and wb21-5 appeared to be 100% identical. The sequences of segment 4 (GP) (212 bp) and segment 5 (NP) (261 bp) were compared with those of the tick-derived OZV EH-8 strain from Ehime Prefecture and the human case-derived OZV Ibaraki/O10-S/2022 strain. The nucleotide sequences of segment 4 (GP) in t23-416 and wb21-5 showed 99.1% identity with OZV EH-8 and 100% identity with OZV Ibaraki/O10-S/2022. Similarly, the nucleotide sequences of segment 5 (NP) showed 98.9% identity with OZV EH-8 and 100% identity with OZV Ibaraki/O10-S/2022. Phylogenetic analyses by ML estimated that both segments of t23-416 and wb21-5 were more closely phylogeny to OZV Ibaraki/O10-S/2022 than to OZV EH-8 (Figure 1). The OZV nucleotide sequences obtained in this study have been deposited in GenBank under accession numbers LC885180–LC885183.

### 3.4. Seroepidemiology Against OZV in Wild Boars

**Figure 2:**
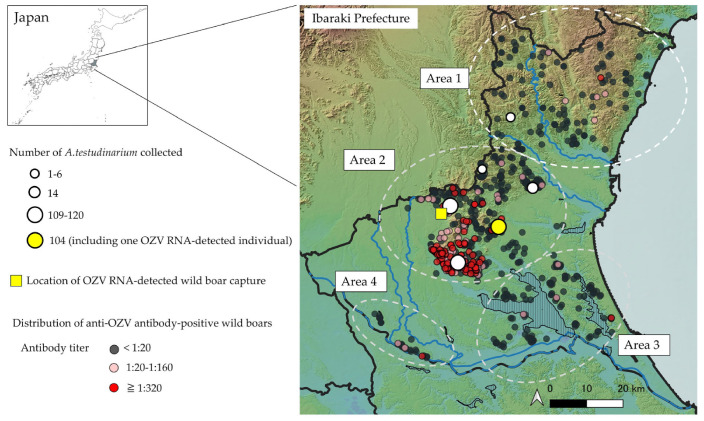
Distribution of *A. testudinarium* and anti-OZV antibody-positive wild boars in Ibaraki Prefecture. The light blue lines indicate rivers, while the vertical lines represent lakes. The number of *A. testudinarium* collected is based on the number of ticks found within a 5 km radius from the center of each plotted circle.

**Figure 3:**
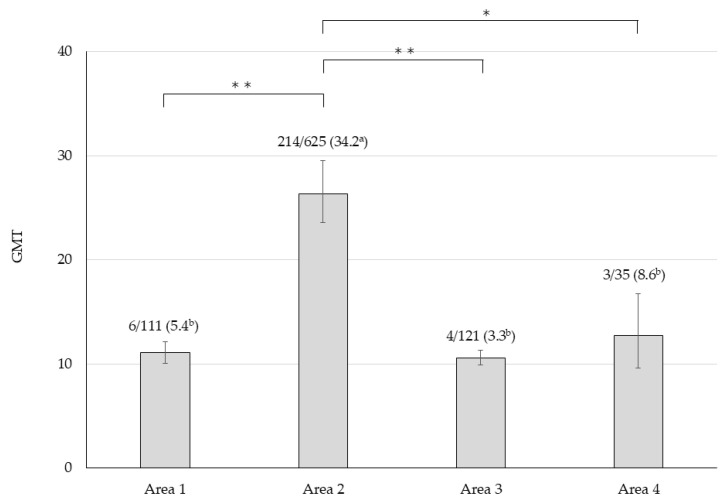
Geometric mean antibody titer (GMT) and antibody positivity rate in each area. Error bars represent the 95% confidence interval of the mean GMT, with the numbers above indicating the number of antibody-positive samples/total tested samples (positivity rate). Asterisks denote significant differences in GMT (*: *p* < 0.05, **: *p* < 0.001), while ^a^ and ^b^ indicate significant differences in antibody positivity rates between groups with different symbols (*p* < 0.05).

The neutralizing antibody titers of the 934 wild boar serum samples were analyzed and summarized by capture year (Table 2). Among these samples, 243 (26.0%) tested positive for OZV antibodies, with GMT of 20.7 (assuming titers ≥1:320 were calculated as 320). Antibody-positive individuals have been continuously detected since 2019. The capture locations of 892 individuals with known origins were plotted on a map of Ibaraki Prefecture (Figure 2). Antibody-positive individuals were distributed throughout the prefecture, with a higher concentration in mountainous areas of the central-western region. To clarify the geographical characteristics of the distribution of antibody-positive individuals, wild boar capture sites were arbitrarily classified into four areas (Area 1–4). Area 1 corresponds to the northern mountainous region, Area 2 to the central region, Area 3 to the southeastern region, and Area 4 to the southern region. The antibody-positive rate and GMT were significantly higher in Area 2 compared to the other areas, while no significant differences were observed among the remaining areas (Figure 3). This region also had a high number of individuals with neutralizing antibody titers ≥1:320. Notably, the wild boar serum sample that tested positive for OZV RNA had a neutralizing antibody titer of ≥1:320.

## 4. Discussion

This study aimed to elucidate the molecular and seroepidemiological characteristics of OZV in Ibaraki Prefecture by collecting ticks, presumed to be vectors, and serum samples from wild boars, considered a potential source of OZV infection. As a result, OZV RNA was detected in both an *A. testudinarium* tick and a wild boar serum, demonstrating for the first time the presence of OZV in the natural environment of this prefecture. Furthermore, seroepidemiological analysis of wild boars suggested that OZV has been actively circulating in the region since 2019. Notably, exposure to OZV among wild boars was prominent in the mountainous areas of the central-western part of the prefecture, indicating the presence of a potential infection hotspot. These results imply that OZV may be maintained in a natural transmission cycle involving ticks and wild boars in Ibaraki Prefecture and could potentially serve as a zoonotic agent.

To date, *A. testudinarium* is the only tick species from which OZV has been detected [1]. In the present study, OZV was also detected exclusively in *A. testudinarium*, despite the examination of seven species from three genera (Table 1), suggesting that this species is a likely natural host of OZV. A tick surveillance conducted in 2021 in Ibaraki Prefecture reported that *A. testudinarium* was distributed from the central to southwestern regions of the prefecture [11]. However, in the present study, *A. testudinarium* was also collected in the northern region, indicating an expansion of its distribution range (Figure 2). *A. testudinarium* is a tick species for which numerous cases of human biting have been reported [12]. Given its high population density in Ibaraki Prefecture, it is considered to be a potential vector for OZV transmission in humans.

Although seropositive animals for members of the *Thogotovirus* genus have been reported worldwide [13,14,15,16,17,18,19], detection of the virus from animal specimens remains limited [20,21,22]. In the present study, the detection of OZV RNA from a wild boar suggests that the virus was causing viremia at the time of sampling. Furthermore, the observed increase in neutralizing antibody titer in the corresponding serum sample indicates that the virus was likely in the process of being cleared from the bloodstream. The discrepancy between relatively high seropositivity rate (26.0%) in the wild boars and the low RNA positive rate (0.11%) may suggest that wild boars may be exposed to OZV but may not sustain enough viremia to serve as active reservoirs. We will need further epidemiological data to clear this issue.

The transmission cycle of OZV had not been elucidated prior to this study, the detection of OZV in both an *A. testudinarium* and a wild boar in the present study may indicate the possibility of viral circulation between these two species. *A. testudinarium* has been reported to exhibit a strong host preference for wild boars, and its distribution in Japan has been shown to correlate with that of wild boars [23]. Furthermore, it has also been identified as the predominant tick species parasitizing wild boars in Japan [24]. These findings support the hypothesis that wild boars are the principal host of *A. testudinarium*, reinforcing the notion that OZV may be circulating between the two.

The OZV strains detected from a tick and a wild boar in this study shared 100% nucleotide identity, suggesting that a single viral lineage is circulating in the natural environment of the prefecture. Furthermore, these strains were also found to be 100% identical to a human-derived virus strain, implying that viruses circulating in nature might be capable of infecting humans.

The strains detected in this study were classified as a different lineage from the EH-8 strain isolated in Ehime Prefecture, demonstrating the genetic diversity among the OZV strain in Japan (Figure 1). Similarly, diversity among strains of Bourbon virus, which is a genetically related virus to OZV, has also been reported within the United States [25], and differences in replication capacity among strains in mammalian cells are believed to contribute to its geographic expansion. To clarify the factors driving the spread of OZV into eastern Japan, further studies are needed to elucidate phenotypic differences among viral strains.

Serological analyses of wild boars revealed that OZV had invaded the prefecture prior to 2019 and had already become established in the local natural environment. In particular, in the mountainous region of central-western Ibaraki Prefecture (Area 2), a high density of individuals with elevated antibody titers was observed, suggesting frequent exposure to OZV in this area (Figure 2). The high antibody titers and seroprevalence in this region indicate a possible high-density distribution of the virus. Moreover, OZV RNA was also detected from both a tick and a wild boar in Area 2, implying that active transmission of the virus is currently ongoing in this region. In contrast, in the other areas, antibody-positive individuals were only sporadically detected, suggesting either low-level viral infiltration in this area or possible migration of the seropositive individuals from other regions with higher prevalence of the virus. Although the typical travel range of the wild boars is within 100 km, travels exceeding 100 km have been reported in certain environmental conditions [26]. Therefore, the movement of wild boars from areas with high viral prevalence could potentially contribute to the spread of OZV into previously unaffected areas with viremic wild boar individuals or infected ticks attached with them.

We did not investigate other wild animals that may serve as hosts for *A. testudinarium*. However, the host range of this tick species has been reported to include not only small and medium-sized mammals but also birds and reptiles, suggesting that a variety of wild animals may contribute to the transmission of OZV [23]. Although previous studies have shown higher seropositivity rates for OZV in sika deer and Asian black bears than in wild boars [2], since the presence of these large mammals has not been confirmed in Ibaraki Prefecture, it is important to investigate smaller vertebrates to better understand the transmission cycle of the virus.

In this study, the high percentage of neutralizing antibodies against OZVin wild boars could be caused by cross-reactivity between the virus and other members of the genus *Thogotovirus*. However, according to previous reports, two Thogotovirus species have been identified in Japan, namely OZVand the Thogotovirus HI-Kamigamo-25 strain, but no cross-reactivity between these viruses has been demonstrated in neutralization tests [27]. In contrast, Bourbon virus that has been shown to cross-react with OZVhas not been detected in Japan [27]. Therefore, although it is possible that cross-reactivity between OZVand as yet unidentified Thogotovirus species in Japan caused an overestimation of the antibody-positive rate, we do not have any candidate virus that could cause such cross-reaction to our knowledge.

This study confirmed the spread of OZV in the natural environment in the Ibaraki prefecture. Although only a single human case of OZV infection has been reported in the prefecture to date, undetected cases of OZV is also possible based on the result in this study. Therefore, diagnostic tests should be considered for detection of OZV infection for patients suspected of having tick-borne diseases or unexplained myocarditis. While this study clarified the exposure history of wild boars to OZV in the prefecture, the risk of transmission to humans remains unclear. Further seroepidemiological investigations targeting residents of the prefecture are needed to more accurately assess the risk of human infection of the virus in the region.

## 5. Conclusions

This study demonstrates the first report ofOZV circulation in the natural environment on the Ibaraki Prefecture, Japan. OZV RNA was detected in both an *A*. *testudinarium* and a wild boar, and serological analysis revealed sustained exposure of the wild boars to the virus. The virus appears to be actively transmitted to the animals in the mountainous central-western region, which may serve as a hotspot. However, relatively high seropositivity rate (26.0%) and the low RNA positive rate (0.11%) in wild boar samples may suggest that the roles of the wild boars as reservoirs in OZV transmission in the prefecture may be limited, though active exposure of the wild boars to the virus was eminent. Phylogenetic analysis also showed that the strains detected in this study were identical to a human-derived strain, suggesting potential zoonotic risk. Although only one human case has been reported in the prefecture so far, the presence of OZV in wildlife and its possible expansion highlight the need for public health awareness. Further studies, including seroepidemiological surveys in humans, is essential to assess the risk of human infection and clarify the viral transmission through its vectors and hosts.

## Figures and Tables

**Table 1:** Number of ticks collected in Ibaraki Prefecture in 2023 by developmental stage (Larva/Nymph/Adult). An asterisk (*) indicates the group that included one nymph positive for OZV RNA.

Tick Species		Larva	Nymph	Adult	Total
*Haemaphysalis*	*f* *lava*	22	1025	189	1236
	*hystrics*	62	136	27	225
	*longicornis*	30	424	9	463
	*m* *egaspinosa*	0	9	1	10
*Amblyomma*	*t* *estudinarium*	108	272 *	2	382
*Ixodes*	*ovatus*	0	5	31	36
	*t* *urdus*	74	34	0	108

**Table 2:** Neutralizing Antibody Titers Against OZV in Wild Boars Captured in Ibaraki Prefecture from 2019 to 2023.

Year of Collection	No. of Samples	Neutralization Antibody Titer						GMT ^†^ (95% CI ^‡^)	Positive Rate (95% CI)
		<1:20 *	1:20	1:40	1:80	1:160	≥1:320		
2019	101	76	2	1	0	5	17	20.4 (15.7–26.6)	24.8 (17.4–34.0)
2020	155	123	3	0	3	7	19	18.3 (15.0–22.3)	20.6 (15.0–27.7)
2021	224	153	5	0	32	16	18	23.6 (19.8–28.2)	32.1 (26.4–38.5)
2022	208	145	6	3	11	16	27	22.6(18.9–27.1)	30.2 (24.4–36.8)
2023	246	194	5	2	4	7	34	18.5 (15.7–21.7)	21.1 (16.5–26.7)
Total	934	691	21	6	50	51	115	20.7 (19.0–22.5)	26.0 (23.3–28.9)

* Sera that did not show an 80% reduction in plaque numbers at the 1:20 dilution was recorded as <1:20. ^†^ Geometric mean antibody titer. ^‡^ Confidence intervals.

## Data Availability

The original contributions presented in this study are included in the article/Supplementary Materials. Further inquiries can be directed to the corresponding authors.

## Supplementary Materials

The following supporting information can be downloaded at: https://www.mdpi.com/article/10.3390/microorganisms13112421/s1, Table S1: List of primers for detection of OZV RNA; Figure S1. Detection sensitivity of the RT-qPCR for OZV RNA; Figure S2. Detection sensitivity of the conventional RT-PCR for OZV RNA.
