# Supplementary material for: Molecular Epidemiology and Seroepidemiology of Oz Virus Infection in Ticks and Wild Boars in Ibaraki Prefecture, Japan
Source: Microorganisms. 2025 Oct 22;13(11):2421. doi: 10.3390/microorganisms13112421 (PMC12654443; doi:10.3390/microorganisms13112421)
Supplement: Supplementary file 1 [file microorganisms-13-02421-s001.zip › microorganisms-3850156-supplementary.pdf]

microorganisms

Table S1. List of primers for detection of OZV RNA. To detect segment 4, primer set [a] was applied to RNA extracted from the RT-qPCR-positive tick specimen, and primer sets [b] were applied to RNA extracted from the RT-qPCR-positive wild boar specimen.

Assay	Segment	Primer Name	Position	Sequence (5′-3′)
conventional RT-PCR	4	segment 4_forward [a]	1140-1538	TGCTGTCACCATGCATATCA
		segment 4_reverse [a]		TGAACACTGCACCACCAATG
		segment 4_ foward_nested_1st [b]	996-1478	TGGCCCAAATAGACCATCGG
		segment 4_ reverse_nested_1st [b]		GCAGTTTCCCAGACACCCAT
		segment 4_ foward_nested_2nd [b]	1103-1354	GACGCAGCCACTACATTCCT
		segment 4_ reverse_nested_2nd [b]		CCAACCCTCCTCGTCTGCTA
	5	segment 5_forward	1055-1355	ACGCTCCATGAACACACCAG
		segment 5_reverse		ACGCTCCATGAACACACCAG

 https://doi.org/10.3390/microorganisms13112421

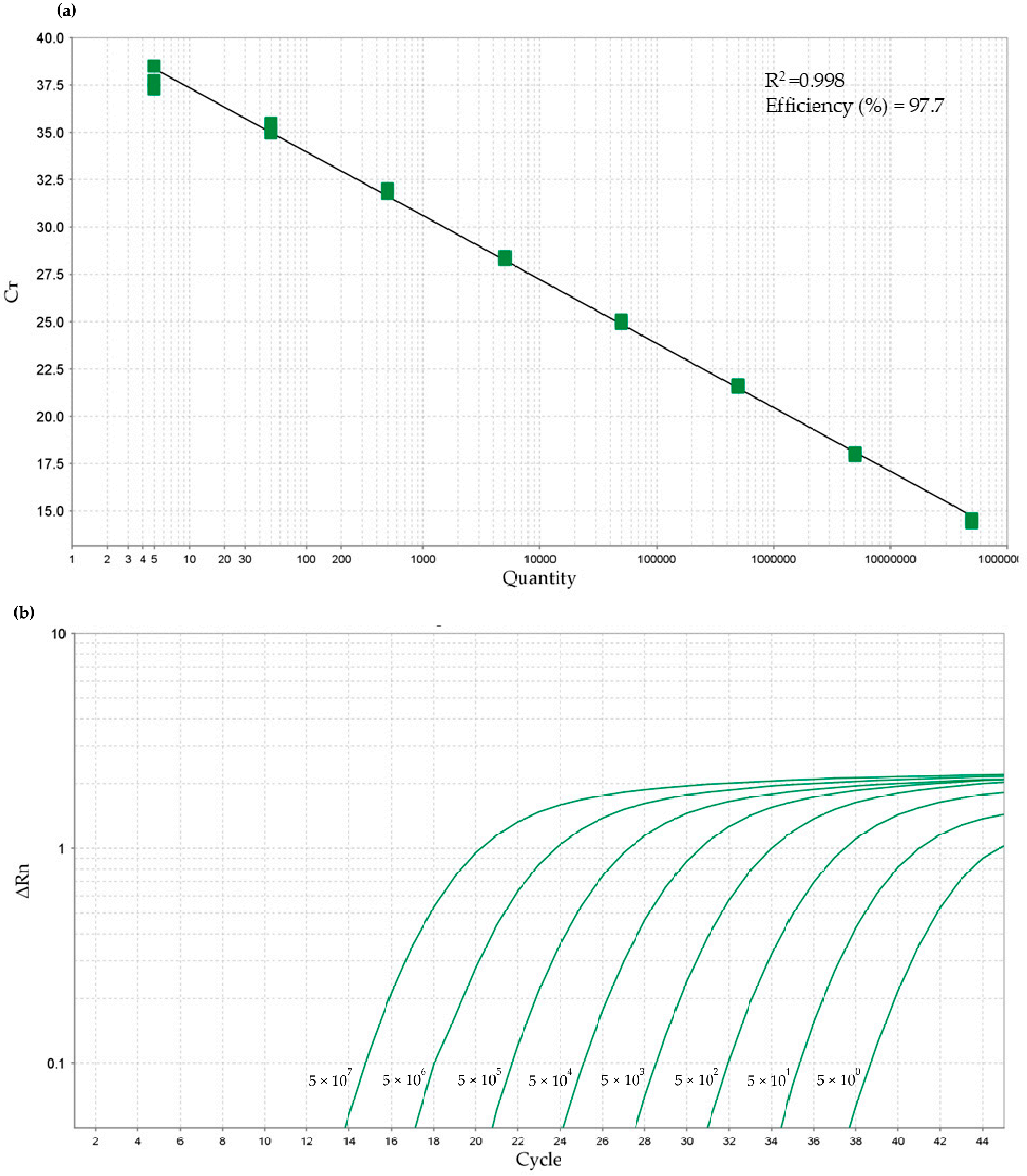

Figure S1. Detection sensitivity of the RT-qPCR for OZV RNA. 10-fold serial dilutions of the full-length synthetic segment 5 RNA, ranging from $5{\times}10^7$ to $5{\times}10^0$ copies per reaction, were prepared. (a) Each dilution series of RNA was tested in triplicate. The *x*-axis indicates the logarithmic concentration ($\log_{10}$) of the RNA and the *y*-axis indicates the C_t value. (b) Each dilution series of RNA was tested in a single replicate. The *x*-axis indicates the C_t value and the *y*-axis indicates ΔRn.

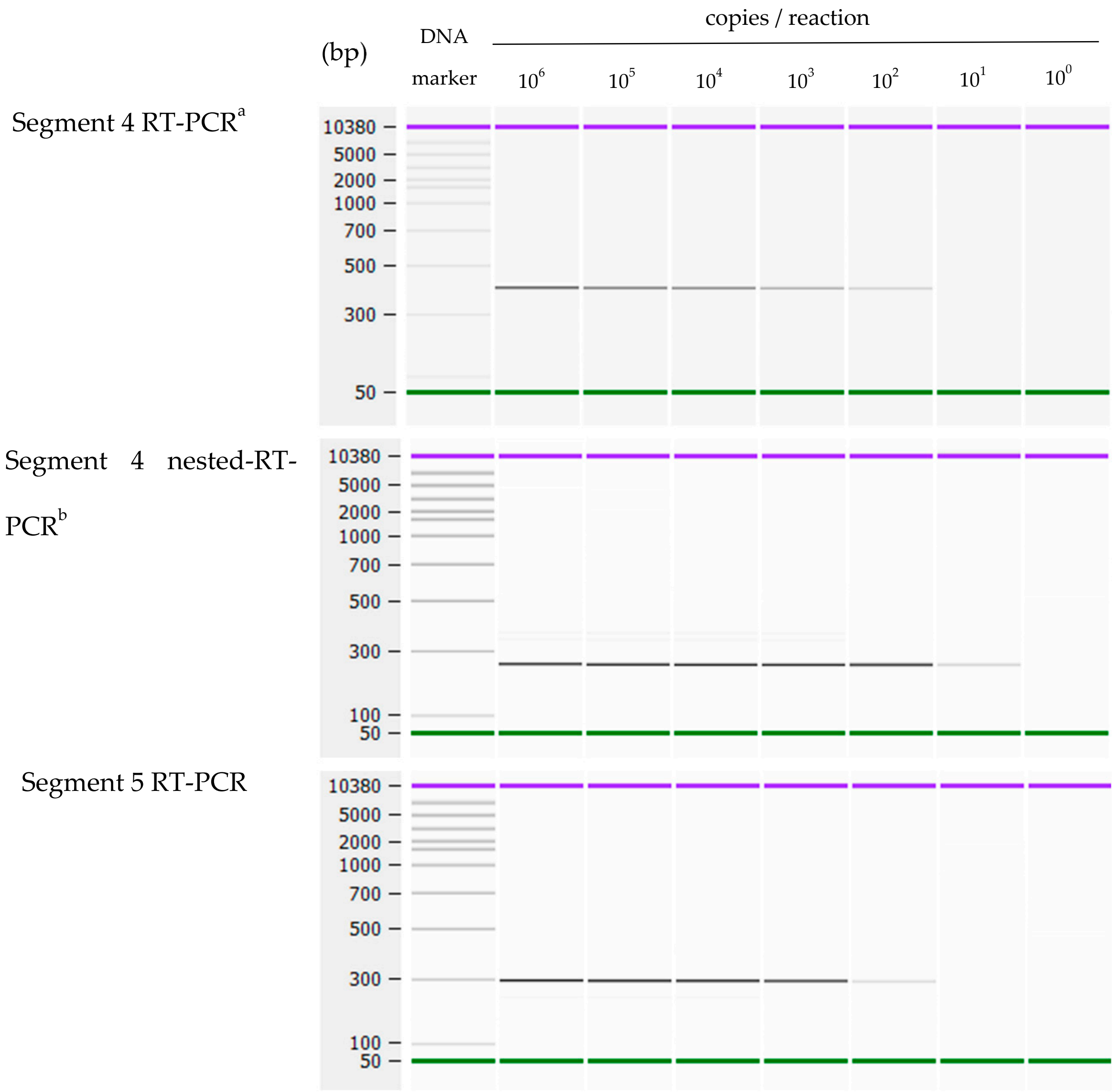

Figure S2. Detection sensitivity of the conventional RT-PCR for OZV RNA. A 10-fold serial dilution of full-length synthetic RNAs for segment 4 and segment 5, ranging from 1×10^6 to 1×10^0 copies per reaction, was prepared. Conventional RT-PCR was performed using the primer sets listed in Table S1, and products were analyzed using a Bioanalyzer.
